# Genetic background modulates behavioral impairments in R6/2 mice and suggests a role for dominant genetic modifiers in Huntington’s disease pathogenesis

**DOI:** 10.1007/s00335-012-9391-5

**Published:** 2012-01-31

**Authors:** Randi-Michelle Cowin, Nghiem Bui, Deanna Graham, Jennie R. Green, Lisa A. Yuva-Paylor, Andreas Weiss, Richard Paylor

**Affiliations:** 1 Department of Molecular and Human Genetics, Baylor College of Medicine, Houston, TX 77030 USA; 2 Novartis Institutes for BioMedical Research, Neuroscience Discovery, Basel, Switzerland; 3 Department of Neuroscience, Baylor College of Medicine, Houston, TX 77030 USA

## Abstract

Variability and modification of the symptoms of Huntington’s disease (HD) are commonly observed in both patient populations and animal models of the disease. Utilizing a stable line of the R6/2 HD mouse model, the present study investigated the role of genetic background in the onset and severity of HD symptoms in a transgenic mouse. R6/2 congenic C57BL/6J and C57BL/6J × DBA/2J F1 (B6D2F1) mice were evaluated for survival and a number of behavioral phenotypes. This study reports that the presence of the DBA/2J allele results in amelioration or exacerbation of several HD-like phenotypes characteristic of the R6/2 mouse model and indicates the presence of dominant genetic modifiers of HD symptoms. This study is the first step in identifying genes that confer natural genetic variation and modify the HD symptoms. This identification may lead to novel targets for treatment and help elucidate the molecular mechanisms of HD pathogenesis.

## Introduction

Huntington’s disease (HD) is a progressive neurodegenerative disorder caused by an expansion of the polyglutamine (Q) tract in the human Huntington’s disease gene *HTT*. HD exhibits complete penetrance with 40Q or more but can occur in individuals with as few as 35Q. The hallmark symptoms of HD include a variety of motor, cognitive, and psychiatric symptoms, but specific phenotypes present with a large degree of heterogeneity. While repeat length is the strongest predictor for age of onset and accounts for ~70% of the age-of-onset variation in HD (Andrew et al. 1993; Brinkman et al. 1997; Duyao et al. 1993; Snell et al. 1993; Stine et al. 1993), little is known about the factors that contribute to the remaining 30% of variability observed. In patients, the strongest correlation for the remaining age-of-onset variation and heterogeneity in psychiatric symptoms is family history (Djousse et al. 2003; Lovestone et al. 1996; Rosenblatt et al. 2001; Squitieri et al. 2000; Telenius et al. 1993; Tsuang et al. 1998, 2000; Wexler et al. 2004), suggesting the influence of genetic and environmental factors beyond the disease gene in the variability of HD onset, symptoms, and progression. Evidence for genetic modification of HD has been found in several patient population studies (Djousse et al. 2004; Gayan et al. 2008; Li et al. 2003, 2006; Metzger et al. 2006a, b, 2008; Taherzadeh-Fard et al. 2009) as well as animal models, including yeast (Giorgini et al. 2005), *Drosophila* (Branco et al. 2008), and mice (Van Raamsdonk et al. 2007).

Although many symptoms of HD can be easily studied in mice (e.g., activity, motor coordination, sensorimotor gating, and anxiety) and are modulated by genetic background (Bolivar et al. 2000; Crawley et al. 1997; Rogers et al. 1999; Spencer et al. 2011; Van Raamsdonk et al. 2007), to date no studies to identify modifiers of HD have been performed using an unbiased screen in a mouse model. The early onset and severity of disease phenotypes make the R6/2 HD mouse model ideal for an unbiased modifier screen (Mangiarini et al. 1996). In a recent study we presented the first characterization of a novel congenic R6/2 line exhibiting a relatively stable polyglutamine expansion (Cowin et al. 2011). Utilizing the advantages provided by this R6/2 line (i.e., pure genetic background, early disease onset, and intergenerational repeat stability), we bred the C57BL/6J (B6) 110Q R6/2 mice to inbred DBA/2J (D2) mice to generate an F1 cross of B6D2F1 (F1) mice. Utilizing this breeding strategy, we assessed whether behavioral responses of B6 R6/2 mice can be altered by the presence of a D2 allele. The findings from the present study indicate that HD-related phenotypes can indeed be modified by a change in genetic background in R6/2 mice and suggest the existence of dominant genetic modifiers that both ameliorate and exacerbate HD-like symptoms.

## Materials and methods

### Animals

C57BL/6J (B6) female mice carrying ovaries from congenic B6 110-polyglutamine (110Q) R6/2 females were obtained from the Jackson Laboratory (Bar Harbor, ME) (Cowin et al. 2011). The ovarian-transferred female mice were bred to male B6 or male DBA2/J (D2) mice creating two lines of R6/2 and wild-type mice: B6 and B6D2F1. Mice were housed two to five per cage with a 12-h light cycle. Food and water were provided ad libitum. Animal care and testing was approved by the Baylor College of Medicine Animal Care and Use Committee and in accordance with NIH guidelines.

### Genotyping and CAG repeat length

Mice were genotyped using PCR amplification of tail DNA. Primers (5′-GCCGCTCAGGTTCTGCTTT-3′ and 5′-AAGGCCTTCATCAGCTTTTCC-3′) were used to amplify the 5′ region of the transgene, yielding a 150-bp product in transgenic samples. Polyglutamine expansion size was monitored in all mice using tissue obtained from ear clips. Laragen, Inc. (Los Angeles, CA, USA) measured polyglutamine length from ear clip DNA using Genescan and sequencing modes on an ABI 377 sequencer. Mice used in this study had repeats ranging from 107 to 120 CAG.

### Morbidity analysis

Mice were monitored daily before noon for symptoms of morbidity. Morbidity was determined when all of the following phenotypes were present: kyphosis, tremor, body weight loss, and immobility. When all these phenotypes were present, the animal was near death (i.e., within 1–3 days), and on some occasions mice were found dead. When morbidity was determined, mice were euthanized. Age at morbidity was recorded and analyzed as percent “survival” using Kaplan–Meier analysis and χ^2^ test for significance. The numbers for each age group tested were as follows: B6, *n* = 48 Tg females, *n* = 60 Tg males; B6D2F1, *n* = 35 Tg females, *n* = 45 Tg males. The repeat range for mice used in this experiment was 108–113 in B6 Tg mice and 107–112 in B6D2F1 Tg mice (Tg = transgenic).

### Behavioral testing

Male and female 110Q mice underwent a battery of behavioral tests performed in the following order: (1) open-field activity, (2) rotarod, (3) prepulse inhibition (PPI), and (4) passive avoidance assay (PA). Mice underwent no more than one test per day and each test was performed with 1–3 days in between. Testing was completed over a 10-day period. Before testing each day, mice were given a 30-min rest period after being moved to the testing room. All experiments were carried out between 8 a.m. and 1 p.m. The numbers for each age group tested were as follows: 8-week-old B6, *n* = 4 Tg females, *n* = 6 Tg males, *n* = 3 Wt females, *n* = 7 Wt males; 8-week-old B6D2F1, *n* = 12 Tg females, *n* = 10 Tg males, *n* = 17 Wt females, *n* = 13 Wt males (Wt = wild-type). Naive mice were used in each group. The average repeat length for mice used in behavioral experiments was 110.10 ± 0.433 SEM for B6 R6/2 mice and 110.36 ± 0.429 SEM for B6D2F1 R6/2 mice.

#### Open-field activity

Mice were placed in the center of a clear Plexiglas arena (40 cm × 40 cm × 30 cm). Testing was 30 min long, during which time mice explored freely. During testing, 800 lx overhead lighting was positioned above the chamber and 55-dB white noise was presented. During testing, the VersaMax Animal Activity Monitoring System (AccuScan Instruments, Inc., Columbus, OH, USA) was used to monitor and record activity as detected by photobeam interruptions. Beam breaks were recorded over 2-min intervals throughout the test period. Data from the entire 30-min test were analyzed in measures of total distance traveled, vertical beam breaks, and distance traveled in the center of the arena (22.5 cm × 22.5 cm). The center distance was divided by the total distance (center:total distance ratio) and used as an index for anxiety-like behaviors (Mathis et al. 1994; Treit and Fundytus 1988).

#### Rotarod

Prior to rotarod testing, the body weight for each mouse was recorded. To measure motor skill and learning, mice were placed on an UGO Basile Accelerating Rota-Rod (Ugo Basile Company, Collegeville, PA, USA) and the latency to fall or lose balance was recorded. If a mouse lost its balance and was not walking on top of the beam but continued to hold on to the rod, the time to the first full revolution was used. The rotation of the rod accelerated from 4 to 40 rpm over a 5-min trial. Mice were given four trials per day over two consecutive days. There was a minimum of 20 min between each trial. Latency to fall (or lose balance but ride around one full revolution) was averaged across all eight trials and analyzed.

#### Prepulse inhibition

Acoustic startle responses to indicate sensorimotor gating performance were measured using the SR-Lab System (San Diego Instruments, San Diego, CA, USA). Mice were placed inside holding tubes and an initial 5-min 70-dB background noise was presented. A test session included six blocks of trials presented in a pseudorandom order. Each block included eight trial types with each trial type presented once; intertrial intervals were 10–20 s. The “startle-only” trial type was a 40-ms 120-dB sound. Prepulses of 4, 8, and 12 dB above the 70-dB background were used. Another three trial types included both prepulse and acoustic startle stimuli. In these trial types, prepulses were 20 ms in duration and were presented 100 ms before the startle stimulus. The last trial type consisted of background noise and was used for measuring baseline movement inside the holding tube. Responses were measured for 65 ms following stimulus presentation. Maximum startle amplitude was used as the dependent variable and mice with startle amplitude less than 100 were excluded from study results.

Percent PPI of a startle response for each prepulse level was calculated as follows:

$$ 100 - [\left( {{\text{startle response on acoustic prepulse}}\; + \; {\text{startle stimulus trials}}/{\text{startle response alone trials}}} \right)\; \times \; 100]. $$

The average PPI of all three prepulse intensities was used as the measure for this experiment.

#### Passive avoidance (PA)

This test was conducted in a two-chamber box (42 cm × 16 cm × 21 cm) (Med Associates, St. Albans, VT, USA) divided by a white partition containing a sliding door. One chamber was clear and brightly lit (approximately 800 lx) while the other was covered and kept dark. Each day of this 3-day test mice were transferred from a cage to the bright chamber and the partition was opened after 10 s. Each day the latency to enter the dark side was recorded, with a maximum of 300 s. On days 1 and 2, after a mouse entered the dark, the partition door was closed and a 2-s 0.75-mA foot shock was administered via a grid floor; after 10 s the mouse was returned to its home cage. Approximately 24 h passed between testing days. Vocalization was noted as an indication that the animal detected the foot shock stimulus.

#### Statistical analyses for behavior

Comparisons were made between genotypes independently at each age. Data for OFA, body weight, acoustic startle response, and PA were analyzed using three-way (genetic background × genotype × gender) analysis of variance (ANOVA). RROD and PPI were analyzed using three-way ANOVA with repeated measures. When interactions were identified, further statistical analyses were performed using simple-effects tests. Analyses revealed changes in phenotypic expression across gender in some assays. For simplicity, all figures represent data from both genders.

### Transgene expression analysis

Mice used in time-resolved (TR-FRET) experiments expressed a mutant transgene with an average of 110 CAG for both lines (B6, 110.83 ± 0.401 SEM; B6D2F1, 109.50 ± 0.992 SEM).

#### Tissue collection

Upon completion of behavior studies, mice were killed by cervical dislocation and whole-brain tissue was immediately removed and stored in Ambion RNA*later* tissue collection RNA stabilization solution (Applied Biosystems/Ambion, Austin, TX) at 4°C for 1–7 days, after which tissue was removed from excess RNA*later* solution and stored at −80°C.

#### Protein isolation and TR-FRET analysis

Crude brain tissue homogenates from 8-week-old B6 Tg (*n* = 6), 8-week-old B6 Wt (*n* = 4), 8-week-old B6D2F1 Tg (*n* = 6), and 8-week-old B6D2F1 Wt (*n* = 4) were prepared as described previously in collaboration with Novartis Pharmaceuticals, Inc. (Cowin et al. 2011). Briefly, tissue was homogenized in 10× w/v lysis buffer [PBS + 1% Triton X-100 + Complete Protease Inhibitor (Roche, Switzerland)]. Five microliters of homogenate and 1 μl detection buffer (50 mM NaH_2_PO_4_, 400 mM NaF, 0.1% BSA, and 0.05% Tween + antibody mix) were mixed to a final antibody concentration of 1 ng 2B7-Tb + 10 ng MW1-d2 and 1 ng 4C9-Tb + 10 ng 4C9-Alexa488 (in each sample) for the quantification of soluble mutant huntingtin (mHTT) and aggregated mHTT, respectively. Samples were analyzed with an EnVision Reader (PerkinElmer, Waltham, MA). The donor fluorophore terbium was excited at 320 nm. After a 100-μs delay, terbium, d2, and Alexa488 emission signals were read out for 200 μs at 620, 665, and 520 nm, respectively. Data are presented as the fold change in signal over background. TR-FRET data were analyzed using one-way ANOVA for a main effect of genetic background. Independent comparisons were made for soluble and aggregated protein measures.

## Results

To determine whether the presence of a D2 allele produced modification of disease phenotypes of HD or progression in the B6D2F1 relative to congenic B6 110Q R6/2 mice, behavioral analysis was performed. Mice of both genetic backgrounds were tested at 8 weeks of age in a variety of experiments to assay amelioration or exacerbation in phenotypes previously identified in pure B6 R6/2 mice, including motor coordination, exploratory activity, anxiety-related responses, sensorimotor gating, learning, and memory as well as soluble and aggregated mHTT protein levels (Cowin et al. 2011).

### B6D2F1 transgenic mice exhibit delayed onset of morbidity relative to R6/2 mice in the B6 genetic background

**Fig. 1:**
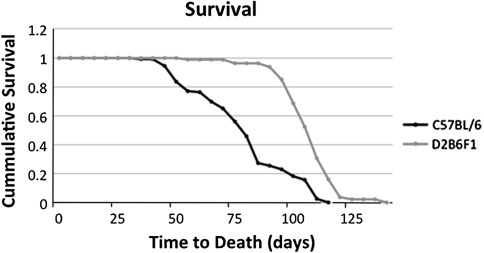
Kaplan–Meier curve of the percent survival of B6 and B6D2F1 transgenic mice over several weeks. B6D2F1 transgenic mice show a significant delay in the onset of morbidity, *P* < 0.001

Mice were monitored daily for signs of end-stage disease phenotypes, including resting tremors, loss of grooming, inactivity, and others (see “Materials and methods” section). Animals that were determined to meet the requirements for morbidity were euthanized immediately in accordance with the guideline set forth by the Baylor College of Medicine Animal Care and Use Committee. The age at which each animal was euthanized was recorded, and differences in percent survival (i.e., morbidity or death) in the two genetic backgrounds were analyzed. We observed a significant delay (χ^2 ^= 69.309, *P* < 0.001; Fig. 1) in the onset of morbidity in the R6/2 F1 mice (107.06 ± 1.38 days) compared to the congenic B6 mice (78.94 ± 2.01 days).

### Presence of the D2 allele modulates activity in wild-type and R6/2 mice, relative to activity levels observed in the B6 background

**Fig. 2:**
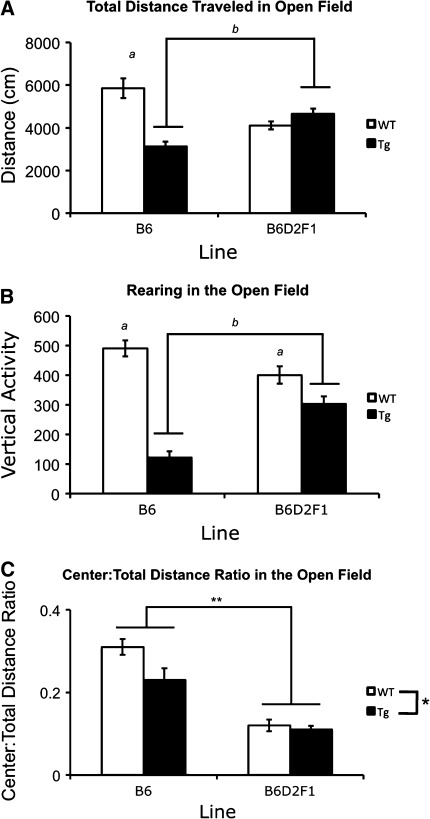
Activity and exploration in the open-field assay is modified in B6D2F1 transgenic mice. **a** Exploratory activity as measured by total distance traveled in an open field. **b** Rearing behavior as measured by vertical activity (beam interruptions). In both (**a**) and (**b**), “*a*” represents transgenic mice that are significantly less active than wild-type littermates and “*b*” represents B6 transgenic mice that are less active than B6D2F1 transgenics. **c** Anxiety-like responses as measured by the center:total distance ratio. *Represents transgenic mice that spend less of their exploration in the center of the open field relative to wild-type mice overall. **Represents B6 mice that spend more time in the center of the open field than B6D2F1 mice overall. All *P* values ≤0.042 by three-way ANOVA and simple-effects analysis

Mice were tested in the open-field assay to assess changes in activity and exploration. Genotype effects were dependent on genetic background and gender as revealed by the significant genetic background × genotype × gender interaction [*F*(1.64) = 4.417, *P* < 0.040]. Follow-up analysis of the interaction revealed that the genotype effect was present in both genders in the B6 background but only in female B6D2F1 mice. Specifically, B6 transgenic mice explored less than B6 wild-type animals at 8 weeks of age (*P* < 0.001) (Fig. 2a). Also, while male F1 transgenics explored in similar fashion to male F1 wild-type littermates (*P* = 0.882), exploration in female F1 R6/2 mice was increased relative to controls (*P* = 0.023). In addition, simple-effects analyses revealed that transgenic F1 mice of both genders explored significantly more than B6 transgenics (*P* ≤ 0.037). Wild-type mice from the F1 line exhibited decreased levels of exploration relative to B6 control mice (*P* ≤ 0.031). A main effect of genotype was also observed [*F*(1.64) = 18.195, *P* < 0.001].

A significant genetic background × genotype interaction [*F*(1.64) = 14.848, *P* < 0.001] was found in open-field rearing activity. Simple-effects analysis revealed that transgenic mice of both genetic backgrounds rear significantly less than littermate controls (*P* < 0.002). In contrast to performance measures of total distance traveled in the open field, the number of vertical beam breaks or bouts of rearing was similar (*P* = 0.180) in wild-type mice from both genetic backgrounds. A clear improvement, however, was observed among transgenics in the B6D2F1 background relative to B6 mutant mice (*P* < 0.001) (Fig. 2b). Rearing behavior in the open field also revealed main effects of genotype [*F*(1.64) = 51.695, *P* < 0.001] and gender [*F*(1.64) = 7.309, *P* = 0.009], with transgenics and female mice exhibiting significantly lower rearing (irrespective of genetic background) than wild-type and male mice, respectively.

### Anxiety-like behaviors in the open field are modulated by genetic background

Anxiety-like behavior in the open field is measured by dividing the distance traveled in the center of field by the total distance traveled in the entire field giving a value for the center:total distance ratio. This measure has been shown to be sensitive to the effects of anxiolytic drugs (Mathis et al. 1994; Treit and Fundytus 1988) suggesting that a lower ratio is indicative of increased anxiety or anxiety-like behaviors. Unlike other measures of the open field assay, no significant interactions were found (*P* ≥ 0.069). However, analysis of center:total distance ratios in the open field did uncover main effects of genetic background [*F*(1.64) = 60.603, *P* < 0.001], genotype [*F*(1.64) = 5.960, *P* = 0.017], and gender [*F*(1.64) = 4.316, *P* = 0.042] (Fig. 2c). Overall, B6D2F1 mice had significantly lower center:total distance ratios than B6 mice. In addition, transgenic mice exhibited lower center:total distance ratios than wild-types and females showed more anxiety-like behavior than male mice.

### Motor impairments on the rotarod assay are exacerbated in B6D2F1 110Q mice

**Fig. 3:**
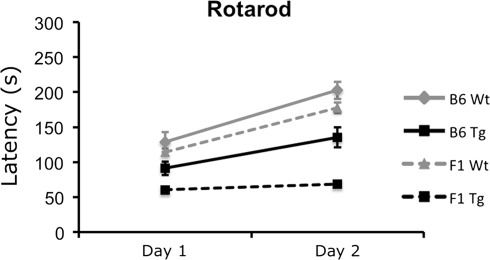
Impaired motor coordination and skill learning on the rotarod is exacerbated in B6D2F1 transgenic mice. Latency to fall from the rotarod was recorded and, in all cases, transgenic mice were impaired relative to wild-type littermates in both genetic backgrounds and on both days of testing (*P* < 0.001). In addition, all genotypes and lines showed significant improvement in rotarod performance between days 1 and 2 (*P* < 0.001). Overall, B6D2F1 mice were impaired relative to B6 mice through the experiment irrespective of genotype (*P* ≤ 0.002)

Differences in performance on the rotarod motor coordination task were observed in 110Q R6/2 mice of different genetic backgrounds and genotypes. Day × genotype [*F*(1.64) = 17.803, *P* < 0.001] and day × genetic background [*F*(1.64) = 4.746, *P* = 0.033] interactions were found. Simple-effects analysis of the significant day × genotype interaction showed that in both B6 and B6D2F1 genetic backgrounds, the latency to fall of transgenic mice was significantly below that of wild-type performance levels on rotarod testing days 1 and 2 (*P* < 0.001). However, there was overall improvement in performance for both transgenic and wild-type mice and mice in both B6 and B6D2F1 backgrounds on day 2 compared to performance on day 1 (*P* < 0.001) (Fig. 3). In addition, the data revealed a worsening of the motor impairment phenotypes in the B6D2F1 background relative to B6 mice on both testing days (*P* ≤ 0.002). A third interaction, genotype × line [*F*(1.64) = 4.906, *P* = 0.030], was also observed. Follow-up analyses showed that in both lines, transgenic mice exhibit significantly less motor skill than control littermates (*P* < 0.001), but it revealed that the increase in rotarod impairments of F1 mice relative to B6 was limited to the transgenic genotype (*P* < 0.001). Wild-type mice performed in a similar fashion on both genetic backgrounds (*P* = 0.062). In addition, the main effects of testing day [*F*(1.64) = 88.455, *P* < 0.001], genetic background [*F*(1.64) = 24.140, *P* < 0.001], and genotype [*F*(1.64) = 80.236, *P* < 0.001] were identified. Specifically, overall performance on day 2 was significantly better than that on day 1, B6D2F1 mice exhibited poorer performance than B6 mice, and transgenic mice were impaired relative to wild-type controls.

### Reduced-weight phenotypes are rescued in B6D2F1 110Q R6/2 mice

**Fig. 4:**
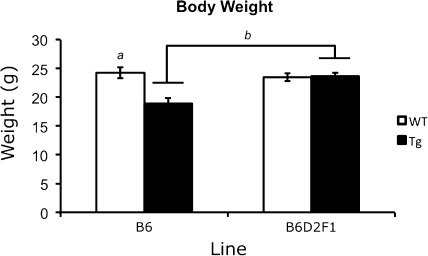
Reduced body weight in transgenic mice is rescued in the B6D2F1 background. “*a*” represents that R6/2 were significantly different from wild types and “*b*” denotes an increase in body weight between B6 and B6D2F1 transgenic mice. All *P* values <0.001 by three-way ANOVA and simple-effects analysis

Both genetic background and genotype strongly influenced body weight in 110Q R6/2 mice. A significant genetic background × genotype interaction [*F*(1.64) = 23.756, *P* < 0.001] was identified and follow-up analyses revealed background-dependent weight phenotypes in transgenics. Specifically, reduced-weight phenotypes were present only in congenic B6 R6/2 mice (*P* < 0.001, Fig. 4). B6D2F1 transgenic mice showed a full rescue of this phenotype, with weight similar to that of control littermates (*P* = 0.787) and significantly more than that of B6 transgenic mice (*P* < 0.001, Fig. 4). These data also revealed main effects of genetic background [*F*(1.109) = 45.979, *P* < 0.001], genotype [*F*(1.109) = 26.544, *P* < 0.001], and gender [*F*(1.64) = 143.824, *P* < 0.001]. Overall body weight of B6D2F1 mice, irrespective of genotype, was higher than that of B6 mice and, not unexpectedly, female mice, irrespective of genetic background or genotype, weighed less than male mice.

### Genetic background modulates acoustic startle responses and PPI but does not alter the phenotypic effects of HD on startle inhibition in R6/2 mice

**Fig. 5:**
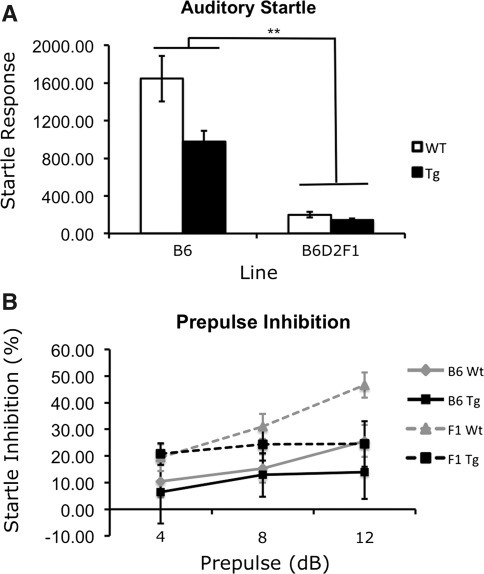
Sensorimotor gating. **a** Acoustic startle response measurements. Both transgenic and wild-type B6D2F1 mice show reduced startle phenotypes relative to B6 mice. **b** Percent PPI of wild-type and transgenic mice at each prepulse. Average PPI was normal in mice in both genetic backgrounds at 8 weeks of age but decreased inhibition was observed by 10 weeks. Overall, B6D2F1 mice show increased inhibition compared to B6 mice. In (**a**) **Reflects a difference in performance between different genetic backgrounds (*P* < 0.001)

Acoustic startle responses revealed a triple interaction of genetic background × genotype × gender [*F*(1.64) = 4.917, *P* = 0.030]. Simple-effects analysis revealed a genotype difference only in male B6 mice. Specifically, B6 R6/2 males exhibited a significantly lower startle response than B6 male wild-type mice (*P* < 0.001). In addition, differences in startle response between the B6 and F1 backgrounds were found for both wild-type and transgenic mice, irrespective of genotype. In all comparisons, F1 mice exhibited a significantly lower startle response than genotype- and gender-matched B6 mice (*P* < 0.001; Fig. 5a). Gender differences were observed, but only in wild-type B6 mice (*P* < 0.001). Main effects of genotype [*F*(1.64) = 13.341, *P* = 0.001], genetic background [*F*(1.64) = 178.035, *P* < 0.001], and gender [*F*(1.64) = 21.230, *P* < 0.001] were also identified. Overall, transgenic, B6D2F1 and female mice exhibited a lower startle amplitude than wild-type, B6, and male mice, respectively.

PPI revealed an overall effect of the prepulse [*F*(2.64) = 6.392, *P* = 0.002] but no interactions with genotype, line, or gender were identified (Fig. 5b). However, a main effect of genetic background was observed [*F*(1.64) = 4.426, *P* = 0.039]. Despite a reduced startle response in the F1 genetic background, B6D2F1 mice exhibited an overall significant increase in percent inhibition relative to B6 mice (Fig. 5b).

### PA performance is affected by genetic background and late-age impairments in transgenic mice are rescued by the D2 allele

**Fig. 6:**
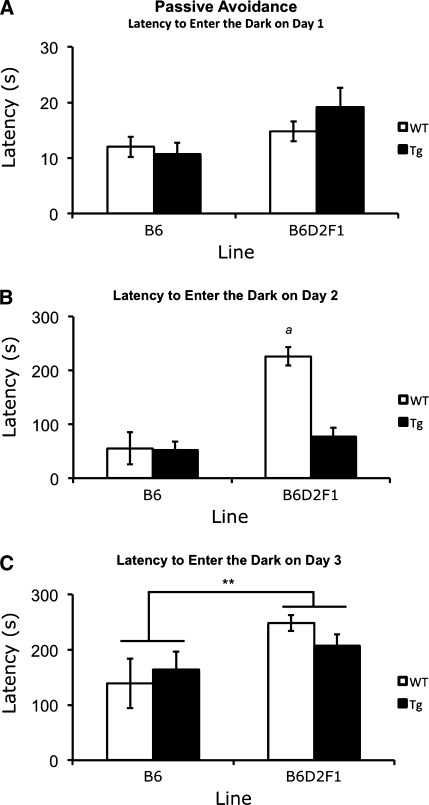
PA learning. **a** Latency to enter the dark on training day 1. **b** Latency to enter the dark on test day 2. **c** Latency to enter the dark on test day 3. No differences in performance between transgenic and wild-type mice were found in either genetic background on day 1. In addition, no significant difference between genetic backgrounds, irrespective of genotype, was identified for day 1 latencies. B6D2F1 transgenic mice exhibited lower latencies to enter the dark than B6D2F1 wild-type mice on day 2, but by day 3 they showed improved learning. In (**b**), “*a*” represents a decreased latency to enter the dark in transgenic mice compared to wild types. In (**c**) **Represents the overall increase in latency in B6D2F1 transgenics compared to B6 transgenic mice. All *P* values ≤0.001 by three-way ANOVA and simple-effects analysis

We previously identified a reduction in PA inhibition in B6 110Q R6/2 mice (Cowin et al. 2011). To investigate whether this phenotype is sensitive to the genetic background, we tested both B6 and F1 lines of R6/2 mice in this striatal-based task (Lorenzini et al. 1995; Prado-Alcala et al. 1985). Performance of naïve mice on training day 1 revealed no significant differences in genetic background, genotype, or gender and no interactions were found (*P* ≥ 0.076; Fig. 6a).

Analysis of day 2 latencies revealed a genetic background × genotype interaction [*F*(1.63) = 10.971, *P* = 0.002]. Simple-effects analysis showed that while mice in the B6 background had no significant difference between wild-type and transgenic performance in day 2 performances for this study (*P* = 0.861), B6D2F1 mice exhibited genotype differences (*P* < 0.001). Specifically, F1 transgenic mice exhibited a shorter latency to enter the dark chamber (Fig. 6b). In addition, data from day 2 show that although transgenic mice from both genetic backgrounds performed in a similar fashion at both ages tested (*P* = 0.413), wild-type mice in the F1 background took significantly longer than B6 wild-type mice to enter the dark (*P* < 0.001). In addition, data from day 2 revealed main effects of both genetic background [*F*(1.63) = 20.031, *P* < 0.001] and genotype [*F*(1.63) = 9.066, *P* = 0.004].

In the PA assay, day 2 serves not only as a test for the learned association of the dark chamber and a foot shock pairing following day 1 of training, but also as a second training day. After entering the dark chamber on day 2, the mice receive a second presentation of the dark chamber—foot shock pairing. Follow-up testing on day 3 is performed to assess learning after two training sessions (foot shocks received on days 1 and 2). In this study, performance on day 3 showed a genetic background × gender interaction [*F*(1.63) = 8.841, *P* = 0.004]. Simple-effects analysis identified differences between genetic backgrounds in female mice. B6D2F1 female mice showed significantly longer latencies than B6 females (*P* < 0.001; Fig. 6c). In addition, an overall effect of genetic background [*F*(1.63) = 12.911, *P* = 0.001] but not genotype was identified, indicating normal learning in the PA assay by day 3 for both B6 and F1 mice in the present study.

### Brains of B6D2F1 transgenic mice have higher levels of soluble mutant protein but not aggregated protein

**Fig. 7:**
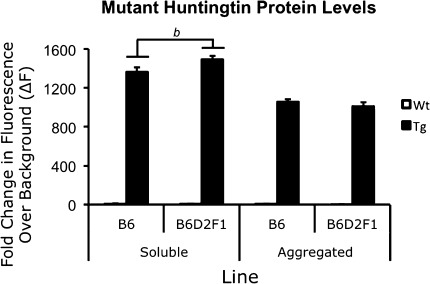
TR-FRET analysis of soluble and aggregated mHTT protein. B6D2F1 mice show increased soluble mHTT but no difference in aggregated protein levels. “*b*” denotes the increased soluble mHTT between B6 and B6D2F1 transgenic mice. All *P* = 0.016 by one-way ANOVA

In addition to investigating a role for genetic background in behavioral phenotype modulation, we analyzed mHTT protein levels in each line of R6/2 mice as a molecular marker of disease using TR-FRET to assess both soluble and aggregated mHTT protein (Weiss et al. 2009). B6 and B6D2F1 mice showed a significant difference in the level of soluble mHTT [*F*(1.8) = 9.215, *P* = 0.016]. Specifically, B6D2F1 mice maintained higher levels of soluble mHTT than B6 transgenics (Fig. 7). This increase was specific to soluble mHTT, however, and genetic background had no effect on the level of aggregated mHTT present [*F*(1.8) = 0.947, *P* = 0.359] (Fig. 7).

## Discussion

Genetic background is known to influence many aspects of behavior in both wild-type and mutant mice (Menalled et al. 2009; Van Raamsdonk et al. 2007). This report presents the effects of genetic background in an F1 study of R6/2 mice carrying 110Q. Mice in both congenic B6 and hybrid B6D2F1 backgrounds were tested in a variety of behavioral tasks known to be impaired in congenic B6 R6/2 mice at 8 weeks old (Cowin et al. 2011). In many of these tasks, the effect of the transgene was dependent on the genetic background, providing evidence for the influence of dominant modifiers of the B6 R6/2 phenotype by the presence of a D2 allele. However, it is important to note that in some assays, the wild-type behavioral responses were significantly different between the B6 and B6D2F1 lines of mice, which was expected given the long history of differences between various inbred strains and F1 hybrid lines (Balogh and Wehner 2003; Logue et al. 1997; Upchurch et al. 1988). These expected differences between wild-type lines might make the interpretation of the effect of the R6/2 transgene on particular phenotypes more challenging. However, the relevant comparisons for the effect of the R6/2 transgene on behavior are with the appropriate control. Abnormal responses due to the presence of the transgene are most suitably evaluated relative to wild-type littermates of the same genetic background. Therefore, we do not believe that differences in wild-type responses between the two lines have significantly confounded the interpretations of the dominant modifier effects observed in the D2B6F1 110Q mice.

In several of the phenotypes tested, the presence of the D2 allele appeared to attenuate the expected R6/2 phenotype based on the response observed in the congenic B6 line. For example, significant delay of morbidity was observed in B6D2F1 transgenic mice. In addition, exploratory activity and body weight were strongly influenced by the presence of the D2 allele.

Activity was dramatically reduced in transgenics of the B6 line, replicating our previous observations (Cowin et al. 2011). In contrast, we found no difference in the amount of exploration between wild-type and transgenic mice in the B6D2F1 hybrid line. In addition, transgenic F1 mice were found to exhibit increased activity relative to B6 transgenics. Taken together, these results suggest attenuation of the reduced-activity phenotype. However, because B6D2F1 wild-type mice exhibited reduced exploration relative to B6 controls, one could argue that the reason the activity of the R6/2 mice on the B6D2F1 background was not significantly impaired relative to their wild-type littermates was because the wild-type exploration was already reduced. While this is certainly possible, we believe close examination of the data do not support this argument. Although the B6D2F1 wild-type behavior was lower than that of the B6 mice, there is still ample opportunity for the B6D2F1 transgenic mice to show further reduced activity. That is, there is little concern for a floor effect, as clearly evidenced by the even further decreased exploration of B6 R6/2 mice. Thus, we believe the interpretation that reduced activity is attenuated with the D2 allele is appropriate. If F1 R6/2 mice were impaired in open-field exploration, the expected result would be that the F1 transgenic mice would show significantly reduced total distance measures relative to the F1 wild-type mice and likely exhibit reduced exploration relative to B6 transgenic mice. Instead, transgenic F1 male performance was indistinguishable from exploration activity in wild-type males on the same genetic background, while female F1 transgenics showed greater exploration than wild-type female mice.

Similarly, the reduced body weight observed in R6/2 110Q mice on the B6 genetic background was ameliorated in R6/2 110Q mice on the B6D2F1 hybrid background. Weight loss is a well-documented characteristic of late-stage disease pathology in HD patients. In the present study, wild-type mice from both B6 and B6D2F1 backgrounds maintained similar weights and reduced-weight phenotypes were observed only in transgenic mice in the B6 background. It is important to note, however, that the delayed morbidity observed in transgenic B6D2F1 mice may result in a delay in the weight-loss phenotype and that this phenotype would be observed at later ages. Although weight loss is among one of the earliest phenotypes to occur in the B6 R6/2 mice (Cowin et al. 2011) and B6D2F1 transgenic mice showed several overt phenotypes by 8 weeks of age, the possibility that the presence of the D2 allele may alter the timeline of this phenotype cannot be excluded in the present study.

However, not all phenotypes tested showed apparent improvement by the presence of a single D2 allele. Exacerbation of impairment was observed in the rotarod motor performance on this assay. Although modification of phenotypes in either direction (i.e., amelioration or enhancement) is expected in any dominant modifier screen, the results of the rotarod assay in this study were surprisingly incongruent with the improvement in transgenic F1 activity measures of the open field. These data clearly show that the motor and activity phenotypes are separable and may be modified by different genes or the same proteins acting in different pathways or cell types.

Still other HD-related symptoms previously observed in congenic B6 R6/2 mice were not altered in transgenic mice. Specifically, anxiety-related behavior, as indicated by the center:total distance ratio in the open-field assay, was found to be generally increased in both transgenic and wild-type B6D2F1 mice relative to B6 animals, but transgenic mice in both backgrounds were found to exhibit more anxiety-like behavior than wild-type littermates. While it is clear that dominant genetic modifiers exist in the D2 genetic background to increase anxiety-like behaviors in wild-type mice (as measured in the open field), the effects that the unknown modifier genes exert on behavior do not alter HD-like symptoms in the R/62 mice and therefore do not appear to interact with the disease mechanism causing the anxiety-like phenotype.

In other assays, the pattern of data was complicated making interpretation more difficult. For example, PPI revealed an overall effect of genetic background in “baseline” or “normal” performance of both wild-type and transgenic animals in the B6D2F1 background. Startle amplitudes were significantly lower in F1 mice of both genotypes relative to B6 mice. Interestingly, despite decreased startle amplitudes, PPI data showed the F1 background to have improved inhibition relative to B6 mice. Again, this pattern of data suggests that any changes due to genetic background modification that are observed between the B6 and F1 lines are limited to dominant effects on baseline behaviors in the presence of a D2 allele and are likely not directly impacting the disease mechanism for this trait. It is important to note that although no dominant modification of the R6/2 transgenic phenotype was identified in the B6D2F1 background for anxiety or sensorimotor gating, these findings do not refute the existence of recessive modifiers or negate the possibility of finding modification in another background strain.

Behavioral phenotypes were not the only measures of disease exhibited by R6/2 mice that showed modulation by the presence of a D2 allele. Soluble protein levels of mHTT were also observed to be altered by genetic background which suggests the presence of dominant modifiers. Interestingly, no modification of aggregated protein levels was observed. Taken together with the delayed morbidity and amelioration of other phenotypes in B6D2F1 mice, the increased levels of soluble protein without similarly increased aggregated protein levels may suggest improved clearance of the mutant protein or simply delayed aggregation of the increased levels of soluble protein.

The present study presents the first data from an ongoing modifier screen for dominant genetic modifiers of HD-like phenotypes in R6/2 mice. The presentation of a single D2 allele in the B6 background showed dominant effects on the R6/2 phenotype, both ameliorating and exacerbating the R6/2 disease phenotypes. While further insights into phenotypes showing amelioration may be gained through additional studies of B6D2F1 mice at later ages, we consider the characterization of the precise age of onset in F1 mice to be of lower priority and less interesting than the search for modifier genes.

Unlike traditional mammalian modifier screens that use ENU mutagenesis to mutate random genes in hopes of identifying novel proteins to further elucidate a known pathway (Beier and Herron 2004; Justice et al. 1999), this study presents dominant modification by the addition of normal, unmutated genes expressed in a different genetic background. An unbiased modifier screen based on genetic variation rather than random mutation is likely to prove extremely valuable in the study of human diseases in which traits or symptoms are strongly hereditary and correlate with family history (i.e., genetic background). Using B6XD2 recombinant inbred strains and mapping QTLs (quantitative trait loci), we hope to identify genes that confer differential and dominant modulation of HD-like phenotypic expression in the R6/2 mouse. Identifying genes that modify the HD symptoms may lead to novel targets for treatment as well as to help elucidate the molecular mechanisms of HD pathogenesis that may eventually lead to a cure.
